# Effects of feeding different proportions of silver leaf desmodium (*Desmodium uncinatum*) with banana (*Musa paradisiaca*) leaf on nutrient utilization in Horro sheep fed a basal diet of natural grass hay

**DOI:** 10.5713/ajas.17.0831

**Published:** 2018-03-02

**Authors:** Diriba Chali, Ajebu Nurfeta, Sandip Banerjee, Lars Olav Eik

**Affiliations:** 1 College of Agriculture, Hawassa University, P. O. Box, 5, Hawassa, Ethiopia; 2 Department of International Environment and Development Studies, Noragric, Norwegian University of Life Sciences, P. O. Box 5003, 1432 Aas, Norway

**Keywords:** Banana Leaf, Body Weight Gain, Feed Intake, Horro Sheep, Silver Leaf Desmodium

## Abstract

**Objective:**

The objective was to evaluate feed intake, digestibility, body weight change and carcass characteristics of sheep fed a basal diet of hay supplemented with banana leaves and silver leaf desmodium.

**Methods:**

Thirty yearling lambs with an average initial body weight of 15.85±1.6 kg were grouped into six blocks of five rams in each block. The treatments were: hay alone (T1), hay+ 100% banana leaf (T2), hay+67% banana leaf+33% desmodium leaf (T3), hay+33% banana leaf+67% desmodium leaf (T4), andhay+100% desmodium leaf (T5). Three hundred grams of treatment diets were offered daily on as fed basis. The feeding and digestibility trials lasted for 84 and 7 days, respectively, followed by carcass evaluation.

**Results:**

The total dry matter (DM) intake for T3, T4, and T5 were greater (p<0.05) than those fed T1 and T2 diets. The lowest (p<0.05) organic matter (OM) intake was recorded in rams reared on T1 diet. The total crude protein (CP) intake was in the following order: T5> T4>T3>T2>T1. Ram lambs receiving supplementary diets had higher (p<0.05) DM, OM, CP, neutral detergent fiber and acid detergent fiber digestibility compared with the control diet. The empty body weight and slaughter weight was highest (p<0.05) in rams receiving T3, T4, and T5 diets. The average daily gain and feed conversion efficiency was highest (p< 0.05) in rams receiving the supplementary diets. The dressing percentage on the basis of hot carcass weight linearly increased with increasing levels of desmodium. Rams reared on supplementary diet had higher (p<0.05) rib eye area compared with the control diet.

**Conclusion:**

In conclusion, when banana leaf is used as a supplement to poor quality grass, better body weight gain was obtained when fed in combination with desmodium.

## INTRODUCTION

Livestock are an important and integral component of agriculture which is the backbone of the Ethiopian economy. This agricultural sub-sector does not only provide valuable sources of animal protein for the ever growing human population, but also contribute to the gross domestic product of the country by providing several export commodities, viz. live animals, meat, hides and skins. However, in spite of large numbers the overall contribution of this sector is far from satisfactory and the overall productivity of the livestock sector is one of the lowest even among the countries of Sub Saharan Africa. Thus, there is a net decline in per capita consumptions of livestock products among the residents of the country. At present, annual per capita consumptions of meat in Ethiopia is estimated to be 8.5 kg which is the lowest among all the neighbouring countries although the country hosts the largest livestock herd in Africa’s continent [1].

In Ethiopia, sheep husbandry is primordially traditional in nature and majority of the animals are reared on natural pasture grazing and crop residues. These feeds are generally deficient in most of the important nutrients. Most of the roughages are generally deficient in protein, energy or some minerals. These roughages if fed alone are by and large unable to support adequate growth and milk production among the ruminants. The ever increasing human population also is resulting in rapid shrinkage of range/pasturelands thereby leading to reduction of land available for grazing and fodder production. It is imperative to improve the utilization of the existing feed resources by identifying some alternative and more nutritious feeds and forages. This nutritionally rich forage provides the rumen micro-organisms with much needed nutrient requirements. This is because the forages with low protein content may impair the rumen functions and thus lead to impairment of feed intake and animal performance.

It is important to bridge the widening gap between the demand and supply of the feed resources. This gap can be bridged through improvement in the availability of feed resources which could be achieved through cultivation of improved forages which are high yielding and adapted to the local conditions. Among several such plants which can be easily cultivated at the farmstead is the silver leaf desmodium (*Desmodium uncinatum* [*D. uncinatum*]) which has been widely promoted in the country as a source of protein [2].

The other feed resources commonly cultivated in the homestead and used as livestock feed are banana waste (leaves, stalks, pseudostem, and peels).The residues of the banana are commonly used as livestock feed especially by the ruminants. In the study area after the harvest of banana fruit, the fresh banana leaves, stalks and pseudostems are chopped and directly fed to the ruminant animals. The banana leaves contain about 14% percent crude protein (CP) [3]. However, banana leaf is poorly degraded compared to the pseudostem [4]. Moreover, the finding of a study by Nurfeta et al [5] indicated that enset (*Ensete ventricosum*) leaf, which is the same family with banana, when fed alone had low intake and digestibility. Therefore, feeding with legume with better digestibility and nutrient content could improve the efficiency of feed utilization.

In general, feeding of legumes to the ruminants can contribute to better utilization of poor quality feeds. The amount of forage legumes needed for effective supplementation (to the poor quality forage) could vary with the quality of the basal diet. There is little information on the feeding values of banana leaf to ruminants when compared to feeding of desmodium (*D. uncinatum* cv. Silver leaf) leaves. Currently, because of their wider availability and distribution, these feeds can be used to bridge the gap in feed supply at times of feed scarcity. However, the nutritive values of these forages in a mixture have not been evaluated for their optimal utilization. Therefore, the objective of the study was to evaluate feed intake, digestibility, body weight change and carcass traits of Horro sheep fed a basal diet of natural pasture hay supplemented with banana leaves and Silver leaf desmodium.

## MATERIALS AND METHODS

### Experimental feeds and feeding

The experiment was conducted at Nedjo town, west Wollega Zone of Oromia National Regional State, Ethiopia. Silver leaf desmodium, banana leaf and natural pasture hay was harvested from the campus of Nedjo Agricultural Technical Vocational Educational Training (ATVET) College, Ethiopia. The natural pasture hay (predominately consisted of Bermuda and Rhodes grass) used in the experiment was harvested from a mixed natural pasture land of the ATVET College at late bloom stage. Desmodium was grown in an already established field under the coffee plant, at the edge of agricultural plots and in areas protected from livestock in the college. Banana leaves were harvested after the collection of fruit of banana. All the feed ingredients i.e desmodium, banana leaves and hay were chopped to an approximate size of 5 cm to facilitate consumption by the animals. The chopped leaves were then shade dried on a plastic sheet to minimize nutrient loss and stored at the experimental site. Hay was offered *ad libitum* (20% refusal). Clean fresh water was available all the time. Three hundred grams of ssupplemental diet was provided per head/day which was determined based on the recommendation by Fitwi and Tadesse [6]. Desmodium and banana leaf meal were offered to the animals in two equal halves at 8:00 am and 13:00 pm daily.

### Experimental animals and their management

Thirty yearling rams of Horro breed with initial body weight of 15.85±1.6 kg (mean±standard deviation) were purchased from the nearby market. Age of the rams was determined by dentition method. The feeding trial lasted for 84 days followed by 10 days of digestibility trial, three days for adaptation and 7 days for data collection. The rams were injected with ivermectin according to the recommended dosage (1 mL/50 kg body weight) subcutaneously for the control of internal and external parasites. All animals were housed in individual pens in a well ventilated concrete floor barn. The pen was equipped with feed trough for separate allocation of hay, supplements and water.

### Experimental design and treatments

The experimental design was randomised complete block design that consists of five treatments (Table 1). The animals were blocked based on their initial body weight into six blocks consisting of five rams per block. The animals were then randomly assigned to the five treatments by making sure that the initially body weight across the treatments were similar.

### Growth trial

The growth trial lasted for 84 days after being quarantined for 14 days followed by 14 days of adaptation to the experimental feed. The feed offered and refused corresponding to each treatment diet for each ram were individually recorded daily throughout the experimental period. Daily feed intake was calculated as the difference between the feeds offered and refused. Representative samples of the feed offered and refused across the different treatments were sampled for nutrient analysis.

Initial body weights of the animals were determined by taking the average of two consecutive weights after overnight fasting. Subsequently the body weights were taken at an interval of 14 days throughout the experimental period. Average daily body weight gain was calculated as the difference between final body weight and initial body weight of the rams divided by the number of feeding days. Feed conversion efficiency was calculated as a ratio of daily body weight gain to daily dry matter (DM) intake.

### Digestibility trial

The digestibility trial was conducted following the feeding trial. The digestibility trial lasted for 10 days. The rams were acclimatized to carrying of the faecal collection bag for 3 days, followed by 7 days of total faeces collection. The faecal output per animal was collected each morning and weighed. Twenty percent of the daily faecal excretion were sampled after thorough mixing and pooled over the experimental period. Daily collected faecal samples were then stored frozen (−20°C). After seven days of total faecal collection period, samples taken daily from each animal were thoroughly mixed and sub- sampled. Feed samples from each feed and refusals from each ram were collected daily during the 7 days of digestibility period. The feed samples were pooled per feed type and refusals per treatment were used for chemical analysis.

### Carcass traits

At the end of the digestibility trial, all the rams were slaughtered to assess influence of treatments on the carcass characteristics. The rams were fasted overnight prior to slaughter. The rams were weighed prior to slaughter and the carotid artery and jugular veins were severed to ensure complete bleeding. Decapitation and skinning of the animals were undertaken followed by removal of the trotters. Then uneviscerated carcass weight was taken after which the organs (heart, lungs, gastrointestinal tract, kidneys, livers, and reproductive organ) were removed and weighed. The eviscerated carcasses were then weighed individually. Dressing percentage was calculated from the hot carcass weight. Dressing percentage was expressed as slaughter weight divided by the empty body weight×100. The edible non-carcass component were blood, heart, liver, kidney, tongue, reticulo- rumen, omaso-abomasums, hind gut (small and large intestine), and internal fat (kidney, pelvic, omental, and mesenteric). The hot carcass weight was estimated after subtracting weights of the head, thorax, abdominal and pelvic cavity contents as well as the trotters.

Gut content was determined by weighing it before and after emptying of the contents to assess the eviscerated weight. Rib eye muscle area was traced by dissecting the carcass between 12th and 13th rib, and the average of the right and the left side measurement was taken as value for rib eye area which was measured using a planimeter.

### Chemical analyses

Samples of feed offered, refusal, and faeces were dried in a forced draft oven at 60°C for 72 hours to constant weight to determine partial DM content. The samples were then ground to pass through a 1 mm sieve and stored pending laboratory analysis. All the feed samples, feed refusals and faeces were analysed for DM, ash, CP content according to the procedures of AOAC [7]. The ash content of the sample was determined by combusting the samples at 550°C for 5 h in a muffle furnace. Nitrogen (N) content was determined using the Kjeldahl method and the CP was calculated as N%×6.25 [7]. The neutral detergent fiber (NDF) contents were analyzed using the method of Van Soest et al [8] whereas the acid detergent fiber (ADF) and acid detergent lignin (ADL) contents were determined according to the method suggested by Van Soest and Robertson [9] using ANKOM filter bag technique (ANKOM Technology Corp., Fairport, NY, USA).

### Statistical analysis

Data were analysed using the general linear model procedure of SAS [10]. Treatment means were compared using Tukey test and the values were considered significant at p<0.05. The model used for data analysis was Y_ij_ = μ+t_i_+b_j_+e_ij_; Where: y_ij_ = response variable; μ = overall mean; t_i_ = ith treatment effect; b_j_ = jth block effect; e_ij_ = random error.

## RESULTS

### Chemical composition of the experimental feeds

The chemical composition of the feeds is presented in Table 2. The ash content of banana leaf was higher than that of hay and desmodium leaf. The CP content of desmodium leaves was higher than that of banana leaf, while the CP content of hay was the lowest. The NDF content of desmodium leaves was the lowest while the highest was for hay. The ADF of hay was higher than that of banana leaves and desmodium hay. The ADL content of banana leaf was higher than of that of hay and desmodium leaf.

### Feed intake

The DM and nutrient intake of sheep fed desmodium and banana leaf as a supplement to grass hay is presented in Table 3. The hay DM intake was the highest (p<0.05) for T1 diet while the lowest (p<0.05) was for rams reared on T5 diet. Among the supplemented groups the hay DM intake for T3 and T4 was higher (p<0.05) than that of T2 and T5. The highest (p<0.05) supplement intake was for T5. The total DM intake for T3, T4, and T5 were greater (p<0.05) than those fed T1 and T2 diet. Rams reared on T1 diet had the lowest DM intake compared with other treatments. There was no significant difference in DM intake among T3, T4, and T5 diets. The lowest (p<0.05) organic matter (OM) intake was for T1 diet. The total CP intake was in the following order: T5>T4>T3>T2>T1.

### Dry matter and nutrient digestibility

The DM and nutrient digestibility of Horro sheep fed silver leaf desmodium and banana leaf as supplement to natural grass hay is shown in Table 4. The supplemented sheep had higher (p<0.05) DM, OM, CP, NDF, and ADL digestibility than the control treatments. There was no significant (p>0.05) difference in the digestibility DM and OM between T3, T4, and T5 diets. The highest digestibility of CP was for T4 and T5 while the lowest (p<0.05) was for T1. The digestibility of NDF for the control treatment was lower (p<0.05) than that of the supplemented group, but the supplemented groups had similar NDF (p>0.05) digestibility. The supplemented groups had significantly higher ADF digestibility than the control except T2.

### Body weight change and feed conversion efficiency

The body weight change, average daily weight gain and feed conversion efficiency are presented in Table 5. The average daily gain for supplemented rams was greater (p<0.05) than the control diet. The feed conversion efficiency was the highest (p<0.05) in rams receiving the supplementary diets compared to the control.

### Carcass characteristics

The carcass traits of the rams reared on different treatment diets are presented in Table 6. The slaughter weight was the highest (p<0.05) in rams reared on T3, T4, and T5 diets while the lowest (p<0.05) was observed for the control group. The empty body weight and hot carcass weight of the rams reared on T4 and T5 was higher (p<0.05) than those reared on T1 and T2, the least weight was recorded for T1 diet. The dressing percentages (DP) on slaughter weight basis for T4 diet was superior (p<0.05) to the rams reared on T1 diet while the values among T2, T3, and T5 diets were intermediate. The DP on the basis of hot carcass weight increased linearly increased from T1 to T5. Rams reared on supplementary diets had higher (p< 0.05) rib eye area when compared to the rams reared on control diet with no differences recorded among the rams receiving different supplementary treatments.

### Edible offal

The weight of the blood was highest (p<0.05) for T3, T4, and T5 diets while the lowest (p<0.05) was for T1 diet (Table 7). The weight of tongue for the rams receiving T3 and T5 diets was grater (p<0.05) than those reared on T1 and T2 diets. The weights of heart and total edible offal’s for the rams receiving T5 diet was higher (p<0.05) than those reared on T1, T2, and T3 diets. Rams reared on T4 diet had heavier testis (p<0.05) than those fed T1, T2, and T5 diets. The weight of kidney for rams reared on T4 diet was greater (p<0.05) than those fed on T1, T2, T3, and T5 diets. Rams receiving T4 and T5 diets had the heaviest (p<0.05) kidney fat while it was the lightest (p< 0.05) was for those reared on T1 diet. The weight of the liver was higher (p<0.05) for the rams reared on the supplementary diets when compared to those receiving the control diet.

## DISCUSSION

### Chemical composition of the experimental feeds

The CP content of the banana leaves in the current study was comparable with the findings (11%) of Katongole et al [4]. Rahma and Huque [11] reported the CP content of the banana leaves to be 8.78% which is lower than the present findings. The difference observed might be attributable to the stage of harvest, the management of the plants and also the variety of the banana as it has been reported by Happi et al [12] that young banana leaves had higher CP content than the mature ones.

The CP content of silver leaf desmodium was comparable with the values (13.45%) reported by Jingura et al [13]. The CP contents were; however, lower than that was reported by Nurfeta et al [5].

The CP content of the hay offered to the experimental animals in the current is in agreement with the value reported (7.9%) by Mogeset al [14], but lower than the result (9.12%) reported by Yinnesu and Nurfeta [15]. The nutritive value of forage could be affected by agroecology and management [16] and season and stage of maturity [17]. The CP content of the feed has to be at least 7% to 7.5% to satisfy the maintenance requirement of the ruminants [18]. However, not only CP content but also adequate DM intake is necessary to optimize animal performance. The CP content of the mixed sward grass hay used in the current study is above the minimum CP value reported by Van Soest [18] as it is well above the minimum requirements of the animals.

### Feed intake

The reduction of hay intake in the rams receiving the supplementary ration (silver leaf desmodium and banana leaves) might be due to the substitution effect of the supplements used. The observations are in agreement with that of Moges et al [14] who reported higher hay intake in rams reared on unsupplemented diets. The higher intake of hay in the rams receiving the control treatment when compared to those reared on supplementary diets might be due to the imbalance of nutrients mainly CP and metabolizable energy in rams fed control diet, which have to increase hay consumption to satisfy their minimum nutrient requirements. The increased total DM intake in the sheep raised on supplementary feeds could be due to the palatability and optimal digestibility of the supplementary (banana leaves and silver leaf desmodium) feeds.

The total CP intake in rams of different dietary groups are in agreement with the findings of Amensisa [19] who reported similar results in Horro rams fed on grass hay diets supplemented with different levels of dried *Vernonia amygdalina* foliage and crushed maize grain mixtures. The results are also in close agreement with Van Soest [18] who reported that dietary protein supplementation increased the supply of nitrogen to the rumen microbes, which is correlated with enhancement of microbial population and efficiency thereby enabling them to increase the rate of breakdown of the digesta.

### Dry matter and nutrient digestibility

Feed supplementation enhances the CP intake of the diet and thereby nutrient digestibility of poor quality basal diet. The findings of this study are similar with the work of Tolera and Sundstøl [20] who reported that the feed digestibility was higher in rams reared on supplementary diet. The lower digestibility of nutrients in the rams raised on the control diet alone (lower CP and high ADF content) may influence the nutrient digestibility [20]. When fed alone, low DM, OM, and CP digestibility of banana leaf was reported by Katongale et al [4] which they attributed to high NDF, ADF, and lignin. The same author also reported low degradability of banana leaf compared with Nakati and sweet potato vines. As cited by the same author, banana leaf was reported to contain tannin which has a lowering effect on rumen digestion.

The higher digestibility of CP in this study in rams receiving the supplementary diet could be due to the higher CP intake when compared to their counterparts receiving only the basal diet which is consistent with the report by Yinnesu and Nurfeta [15]. The findings are also in close agreement with those of Tolera and Sundstøl [20] who reported higher digestibility of CP of legume based supplementation, which may be due to the substantial improvement in the rumen microbial activity, especially that of cellulolytic organisms and higher fragility of the cell-walls of the legume supplemented diets.

The digestibility of ADF in sheep receiving T2 (only banana leaves) diets were low and comparable with the findings of Marie-Magdeleine et al [21] which might be due to high tannin content of the banana leaves. Similarly, Nurfeta et al [22] also reported lower DM digestibility in rams reared on enset (*Ensete ventricosum*) leaf, which is of the same family with banana, based diet which they attributed to higher levels of NDF, ADF and lignin in enset leaf. The findings are also consistent with the findings of Pieltain et al [23] who indicated the low digestibility of banana leaves due to high content of anti nutritional factors. As cited by Marie-Magdeleine et al [21] banana leaf contains other anti-nutritional substances such as terpenoids (mainly saponosides) and flavoids which could negatively affect digestibility.

### Body weight change and feed conversion efficiency

The findings of this study indicate that supplementary diet containing the supplements was higher in average daily gain (ADG) than those of natural pasture based diet. This may be due to improvement in DM, OM, and CP intakes and digestibility which can influence the live weight gain, and feed conversion efficiency. In an experiment by Katongale et al [4] sheep fed banana leaf as a sole diet lost body weight compared with sweet potato vines indicating the necessity of feeding banana leaf with other feeds like desmodium for better performance. The rams receiving the supplementary diet had higher body weight gain and nutrient intake. The enhanced gain can be due to better nutritional quality of the supplementary diet particularly high CP content of the feed. Supplemented rations also provide essential nutrients for rumen microorganisms, enhancing the ruminal microorganism and providing nutrients for the sheep [18].

The weight gain observed in this study is similar with those of Garcia et al [24] who reported that banana leaf meal (leaves chopped and sun dried) fed up to 40% on a DM basis improved daily weight gains and feed efficiency of Zebu cattle and sheep alike.

### Carcass characteristics

The higher slaughter weight in the supplemented treatments in the current study is consistent with the result of Alemu et al [25] in goats fed different supplements. The DP of the rams reared on different dietary treatments are comparable with the previous study by Gebresilassie [26] who reported that the DP varied between 48.6% to 50%. The eviscerated DP of the rams reared under different treatments were, however, lower than those reported by Yinessu and Nurfeta [15]. The differences in DP might be attributable to the breed of the rams and also the type of feed available to them [27]. Differences in dressing percentage across the treatments can be attributable to differences in tissue development of the rams reared on higher planes of nutrition [28].

The effect of supplementation was correlated with rib-eye muscle area which means that supplementation resulted in better muscle development. According to the study of Melaku and Betsha [29] higher rib eye area is associated with a higher production of lean meat in the carcass and higher lean/bone ratio. The effect is similar to those of the present study where the rams reared on supplementary diet improved weight gain and also better carcass yield of the rams.

### Edible offal

The greater total edible offal in the supplemented group compared with the control diet could be due to the provision of more nutrients from the supplements. The quality and quantity of the offal/giblets depend on the nutritional status of the animals and their slaughter weight [30]. The findings of the present study are in agreement with Abadi [31] who reported relatively lower total edible offal components in animals reared on control diet when compared with those of the supplemented groups which might be attributable to the poor tissue development in rams receiving the control diet. The high kidney fat observed for the supplemented group in the current study is consistent with the observation by Tafa et al [32] which they have attributed to the supply of more energy from the supplements which may have promoted more fat deposition compared with the control. The developments of the internal organs correlate with the diet received and are in general proportional to the body weight at slaughter.

## CONCLUSION

Banana leaf alone resulted in improvement in intake (DM, OM, and CP) and digestibility but better intake and digestibility was observed when fed in combination with desmodium similar to the level where desmodium alone is used as a supplement. Rams reared on supplementary diets had better average daily gain when compared to the rams reared on control diet with no differences in ADG recorded in rams receiving different supplementary treatments. On the other hand, rams fed on banana leaf alone and in combination with silver leaf desmodium to the basal diet of grass hay improved weight gain and carcass characteristics similar to feeding desmodium alone. In conclusion when banana leaf is used as a supplement to poor quality grass, better body weight gain is obtained when fed in combination with desmodium in sheep feeding.

## Figures and Tables

**Table 1:** Proportion of the treatment diets

Treatments	Proportion of feeds			

	Grass hay	Desmodium leaf (%)	Banana leaf (%)	Daily offer (g) (as fed basis)
1	*Ad libtum*	-	-	-
2	*Ad libtum*	0	100	300
3	*Ad libtum*	33	67	300
4	*Ad libtum*	67	33	300
5	*Ad libtum*	100	0	300

**Table 2:** Chemical composition (% DM unless specified) of experimental feeds

Feeds	Chemical composition					

	DM%	Ash	CP	NDF	ADF	ADL
Hay	95.8	11.1	7.9	70.5	43.7	5.5
Banana leaf	94.4	15.5	11.2	65.7	38	13.5
67% banana leaf:33% desmodium leaf	95.6	14.1	12	63.5	38.2	11.9
33% banana leaf:67% desmodium leaf	93.8	12.7	12.7	61.1	38.3	10.2
Desmodium leaf	94.6	11.3	13.5	58.9	38.5	8.5

DM, dry matter; CP, crude protein; NDF, neutral detergent fiber; ADF, acid detergent fiber; ADL, acid detergent lignin.

**Table 3:** Daily dry matters and nutrient intake of Horro sheep fed silver leaf desmodium with banana leaf as supplement to a basal diet of hay

Intake (g/d)	Treatments1)					SL	SEM

	T1	T2	T3	T4	T5		
Dry matter							
Hay	716^a^	666^c^	692^b^	687^b^	656^d^	***	8.2
Supplement	0	164^b^	175^b^	174^b^	216^a^	**	12.3
Total	716^c^	830^b^	867^a^	870^a^	871^a^	***	13.9
Organic matter	584^b^	714^a^	715^a^	715^a^	715^a^	***	1.7
Crude protein	59.0^e^	81.6^d^	84.7^c^	86.0^b^	89.0^a^	***	0.7
Neutral detergent fiber	498^c^	593^a^	587^a^	567^b^	568^b^	***	9.6
Acid detergent fiber	307^c^	367^a^	361^a^	343^b^	157^d^	***	6.2
Acid detergent lignin	38.5^d^	61.3^a^	58.5^a^	55.5^b^	46.8^c^	***	1.7

SL, significant level; SEM, standard error of mean.

1) T1 = hay alone; T2 = hay+100% banana leaf; T3 = hay+67% banana leaf+33% desmodium leaf; T4 = hay+33% banana leaf+67% desmodium leaf; T5 = hay+100% desmodium leaf.

a,b,c,d p<0.05 value across a row are different.

**Table 4:** The dry matter and nutrient digestibility of Horro sheep fed silver leaf desmodium and banana leaf as supplement to a basal diet of natural grass hay

Digestibility coefficient	Treatment1)					SEM	SL

	T1	T2	T3	T4	T5		
Dry matter	0.58^c^	0.73^b^	0.76^a^	0.78^a^	0.78^a^	0.019	***
Organic matter	0.61^c^	0.77^b^	0.8^a^	0.81^a^	0.8^a^	0.014	***
Crude protein	0.69^c^	0.76^b^	0.78^b^	0.86^a^	0.88^a^	0.024	***
Neutral detergent fiber	0.60^b^	0.77^a^	0.78^a^	0.79^a^	0.77^a^	0.021	***
Acid detergent fiber	0.54^b^	0.36^c^	0.74^a^	0.76^a^	0.76^a^	0.025	***

SEM, standard error of mean; SL, significant level.

1) T1 = hay alone; T2 = hay+100% banana leaf; T3 = hay+67% banana leaf+33% desmodium leaf; T4 = hay+33% banana leaf+67% desmodium leaf; T5 = hay+100% desmodium leaf.

a,b,c p<0.05 value across a row are different.

**Table 5:** Body weight parameters and feed conversion efficiency of Horro sheep fed silver leaf desmodium and banana leaf as supplement to the basal diet of natural grass hay

Parameter	Treatment1)					SL	SEM

	T1	T2	T3	T4	T5		
Initial body weight (kg)	15.6	16.0	15.8	16.2	15.8	ns	0.56
Final body weight (kg)	17.4^c^	19.7^b^	19.7^b^	20.3^a^	19.8^ab^	***	0.34
Body weight change (kg)	1.6^b^	3.8^a^	3.8^a^	4.5^a^	3.9^a^	***	0.33
Average daily gain (g)	17.3^b^	42.5^a^	42.7^a^	49.5^a^	43.5^a^	***	3.7
Feed conversion efficiency (%)	2.6^b^	6.3^a^	6.2^a^	7.0^a^	6.8^a^	**	0.005

SL, significant level; SEM, standard error of mean; ns, not significant.

1) T1 = hay alone; T2 = hay+100% banana leaf; T3 = hay+67% banana leaf+33% desmodiumleaf; T4 = hay+33% banana leaf+67% desmodium leaf; T5 = hay+100% desmodium leaf.

a,b,c p<0.05 value across a row are different.

**Table 6:** Carcass characteristics of Horro sheep fed silver leaf desmodium and banana leaf as supplement to a basal diet of natural grass hay

Parameter	Treatments1)					SL	SEM

	T1	T2	T3	T4	T5		
Slaughter weight (kg)	16.6^c^	18.0^b^	19.3^a^	19.7^a^	20.0^a^	***	0.84
Empty body weight (kg)	14.1^c^	15.2^b^	16.2^ab^	16.8^a^	17.0^a^	***	0.79
Hot carcass (kg)	6.40^c^	7.20^b^	8.40^ab^	8.90^a^	9.20^a^	**	1.08
Dressing percentage							
Slaughter weight base (%)	37.6^b^	42.7^ab^	43.6^ab^	44.8^a^	46.2^ab^	***	4
Empty body weight base (%)	38.6^e^	40.6^d^	43.4^c^	45.8^b^	54.3^a^	***	4.5
Rib eye area (cm^2^)	5.30^b^	6.30^a^	6.40^a^	6.70^a^	6.70^a^	***	0.43

SL, significant level; SEM, standard error of mean.

1) T1 = hay alone; T2 = hay+100% banana leaf; T3 = hay+67% banana leaf+33% desmodium leaf; T4 = hay+33% banana leaf+67% desmodium leaf; T5 = hay+100% desmodium leaf.

a,b,c,d p<0.05 value across a row are different.

**Table 7:** Edible offal (g) of Horro sheep fed silver leaf desmodium and banana leaf as supplement to a basal diet of natural grass hay

Parameters	Treatments1)					SL	SEM

	T1	T2	T3	T4	T5		
Blood	967^c^	1,030^b^	1,175^a^	1,208^a^	1,141^a^	***	8.5
Tongue	77.6^c^	85.8^bc^	97.5^a^	92.8^ab^	101^a^	**	9.19
Testis	213^d^	226.5^cd^	276.2^ab^	299.4^a^	250.5^bc^	***	5.1
Kidney	102^b^	108^b^	106^b^	112^a^	106^b^	**	3.79
Kidney fat	18.5^c^	23.5^b^	26^ab^	28.5^a^	27.5^a^	***	2.79
Empty gut	1,543^b^	1,630^ab^	1,645^ab^	1,758^a^	1,700^a^	*	6.5
Heart	101^c^	107^c^	121^b^	130^ab^	136^a^	***	7.43
Liver	283^b^	311^a^	324^a^	338^a^	311^a^	*	22.2
Tail	267^d^	368^c^	520^b^	701^a^	634^a^	***	78.9
Total edible offals	3,571^d^	3,889.8^c^	4,285.7^b^	4,667.7^a^	4,407^ab^	***	226.6

SL, significant level; SEM, standard error of mean.

1) T1 = hay alone; T2 = hay+100% banana leaf; T3 = hay+67% banana leaf+33% desmodium leaf; T4 = hay+33% banana leaf+67% desmodium leaf; T5 = hay+100% desmodium leaf.

a,b,c,d p<0.05 value across a row are different.
